# A deep learning based dual encoder–decoder framework for anatomical structure segmentation in chest X-ray images

**DOI:** 10.1038/s41598-023-27815-w

**Published:** 2023-01-16

**Authors:** Ihsan Ullah, Farman Ali, Babar Shah, Shaker El-Sappagh, Tamer Abuhmed, Sang Hyun Park

**Affiliations:** 1 Department of Robotics and Mechatronics Engineering, Daegu Gyeonbuk Institute of Science and Engineering (DGIST), Daegu, 42988 South Korea; 2 Department of Computer Science and Engineering, School of Convergence, College of Computing and Informatics, Sungkyunkwan University, Seoul, 03063 South Korea; 3 College of Technological Innovation, Zayed University, Dubai, 19282 United Arab Emirates; 4 Faculty of Computer Science and Engineering, Galala University, Suez, 435611 Egypt; 5 Information Systems Department, Faculty of Computers and Artificial Intelligence, Benha University, Banha, 13518 Egypt; 6 Department of Computer Science and Engineering, College of Computing and Informatics, Sungkyunkwan University, Suwon, 16419 South Korea

**Keywords:** Machine learning, Image processing

## Abstract

Automated multi-organ segmentation plays an essential part in the computer-aided diagnostic (CAD) of chest X-ray fluoroscopy. However, developing a CAD system for the anatomical structure segmentation remains challenging due to several indistinct structures, variations in the anatomical structure shape among different individuals, the presence of medical tools, such as pacemakers and catheters, and various artifacts in the chest radiographic images. In this paper, we propose a robust deep learning segmentation framework for the anatomical structure in chest radiographs that utilizes a dual encoder–decoder convolutional neural network (CNN). The first network in the dual encoder–decoder structure effectively utilizes a pre-trained VGG19 as an encoder for the segmentation task. The pre-trained encoder output is fed into the squeeze-and-excitation (SE) to boost the network’s representation power, which enables it to perform dynamic channel-wise feature calibrations. The calibrated features are efficiently passed into the first decoder to generate the mask. We integrated the generated mask with the input image and passed it through a second encoder–decoder network with the recurrent residual blocks and an attention the gate module to capture the additional contextual features and improve the segmentation of the smaller regions. Three public chest X-ray datasets are used to evaluate the proposed method for multi-organs segmentation, such as the heart, lungs, and clavicles, and single-organ segmentation, which include only lungs. The results from the experiment show that our proposed technique outperformed the existing multi-class and single-class segmentation methods.

## Introduction

Chest X-rays are mostly used to examine the chest’s anatomical structures, such as the lungs, heart, and clavicles for various pulmonary and cardiac disorders. In the healthcare industry, 3.6 billion chest X-rays are performed each year to evaluate the patients’ health conditions^1^. With the increasing amount of chest X-rays, computer-aided diagnostic (CAD) systems can play a significant role to identify chest diseases. Significant research has recently been conducted in CAD systems to facilitate pulmonologists/radiologists to assess chest radiographs. The CAD considers a range of analytical tasks that demand precise segmentation of the anatomical structures in the chest radiography images. These tasks include disease prediction and various size measurements in the chest radiographs, such as determining the existence of pulmonary nodules^2^ or lung disease^3^ using lung field segmentation. Furthermore, cardiomegaly^4^ can be predicted using heart segmentation in chest radiographs. The information about the clavicle position can be used to overcome false positive results and identify the lesions behind a clavicle more consistently.

However, evaluating chest radiographic images is still challenging for indistinct and overlapping body structure borders, such as the lungs, heart, and clavicles. Furthermore, anatomical structures vary in shapes and sizes depending on the patient’s gender, age, and physique. Also, the presence of medical equipment, which includes pacemakers and guidewires, and various artifacts in chest radiography make the segmentation of anatomical structures problematic. Despite these difficulties, significant development has occurred in recent years regarding the improvement of the segmentation methods^4–7^. The researchers have been able to test various segmentation approaches that employ the Japanese Society of Radiological Technology (JSRT) dataset^8^. These approaches include rule-based systems, such as the thresholding of intensity^9^, edge detection-based methods^10,11^, hybrid models^12,13^, and landmark-based models^14,15^. However, these methods require optimal parameter settings for good performance, and they frequently fail when the anatomical structures overlap.

Due to the excellent performance of the CNNs in segmentation tasks, which are notably in biomedical imaging, the CNN-based approaches^5,16^ have quickly gained popularity. Moreover, deep learning methods, such as U-Net have substantially improved segmentation in medical applications, which include vascular segmentation^17^, catheter segmentation in X-rays and realistic images^18–20^, and the lungs segmentation in chest radiography^21^. However, the interest region in the medical images can have similar appearances, which makes it difficult to segment them using U-Net^22^. Deeper CNN structures with more layers are preferred for better feature representation to achieve a higher segmentation performance. Therefore, we utilized a dual CNN architecture with more layers to achieve a better feature representation for the anatomical structure segmentation.

This paper presents a novel segmentation framework that uses a dual encoder–decoder CNN structure with a pre-trained network weights for the chest anatomical structure segmentation in the X-ray fluoroscopic images. The first encoder–decoder network predicts the initial mask using the VGG19 pre-trained network as an encoder. The initially predicted mask is coupled with the input image, and it is fed into a second encoder–decoder network, which comprises of the recurrent residual convolutional layers and an attention gate module to predict the final improved segmentation mask and capture the additional contextual features. The proposed framework is evaluated using three standard benchmark datasets. The JSRT dataset is utilized for three-class segmentation, which contains the data about heart, clavicles, and lungs. The MCCXR and SCXR datasets are employed for single-class segmentation, which includes the lungs only. The proposed method outperforms the existing state-of-the-art (SOTA) CNN architectures for the anatomical structures segmentation in the chest X-rays. The following are the significant contributions of this study.

- We propose a novel dual encoder–decoder architecture to effectively segment the anatomical structures in chest X-ray images. The proposed method can accurately segment both the more prominent structures (lungs and heart) and the smaller structures (clavicles) in chest X-rays.
- Instead of previous methods requiring post-processing^23^ or multi-stage^5^, we employed an end-to-end CNN architecture with efficient training and high accuracy to improve the model sensitivity to foreground pixels without requiring complex heuristics.
- The proposed method incorporates Attention Gating Modules (AGMs) to allow the model to focus on the regions of interest while maintaining the spatial resolution and improving the quality of the feature maps.
- We extensively evaluated three public datasets for the anatomical structure segmentation, that includes multiclass segmentation and single-class segmentation. The experimental results show that our proposed method outperforms the previous SOTA methods on single class anatomical structure segmentation as well as multiclass anatomical structure segmentation.

The rest of the manuscript is arranged as follows. “Related work” presents the prior studies about the anatomical structure segmentation for the X-ray fluoroscopic images. “Proposed method” describes the proposed method in detail. We evaluate the proposed method extensively in “Experiments and results”. “Discussion” provides a discussion about the proposed framework. Finally, “Conclusion” presents the conclusion of the proposed method.

## Related work

In this section, we present the recent advancements in the anatomical structure segmentation in three domains, which include the conventional, shallow learning, and deep learning methods.

### Conventional methods

The conventional feature-based methods employ heuristics algorithms relying on low-level image features. For example, Cheng et al.^24^ locate lung areas by evaluating the Horizontal and Vertical profiles (HVP) of a chest X-ray image. After that, the lungs boundaries are detected using static global intensity thresholding and smoothing operations. Armato et al.^25^first construct a range of threshold values using a histogram analysis instead of utilizing the threshold of static global, which they later apply global thresholding to distinguish the initial lung area, and then use local thresholding to refine the initial segmented lung region. Nevertheless, iterative thresholding makes the process computationally expensive. Furthermore, these techniques^24,25^ produce lower performances when large deformation occurs in the chest region.

Also, these methods are sensitive to image intensities. It is challenging to define a proper threshold value for the accurate segmentation in the X-ray images due to poor contrast^24,25^. Li et al.^10^ employed the first derivative of the HVP to define the initial boundary of the lung area, which was succeeded by an adjustment of iterative boundary and edge tracing. However, this method fails to segment the complicated anatomical structures, such as mediastinum and hemidiaphragm. Iakovidis et al.^11^ utilized a similar approach^10^ to identify the lung boundaries in chest X-ray images using curve interpolation^26^. Xu et al.^27^ introduced an improved method to identify the anatomical structures in chest X-ray images by performing a gradient analysis using the structural relationships among the anatomic landmark positions. After that, the polynomial functions are utilized to smoothen the boundaries of the anatomical structures. Ahmad et al.^28^ employed c-mean clustering (CMC) to improve lung borders. However, these techniques do not need any prior understanding of lung anatomy. Several approaches^10,11,27,28^ are still noise-sensitive, and the results of pre-processing noise filtering with a Gaussian are contingent on the filter size and threshold.

### Shallow learning methods

The shallow learning techniques are considerably sensitive to the feature extraction process. However, the major limitation of these approaches is to select the suitable attributes. Several shallow learning-based frameworks for anatomical structure classification are described in the prior literature. To identify the lung area, Gray et al.^29^ used a multi-layer neural network (MLP) with the k Nearest Neighbor (kNN) and the Linear Discriminant Analysis (LDA) classifiers, which were trained using a variety of local textures, grey levels, and local difference based features. Tsujii et al.^30^ used a Hybrid Adaptive Neural Network (HANN), which includes features, such as adjacent pixel locations, normalized intensity, and histogram equalized entropy. The hidden layers in the HANN are dynamically settled, and thus they can be trained efficiently without over-fitting. Nevertheless, these techniques perform poorly with similarly intensity regions, low contrast, and tilted images.

The Markov random field (MRF) model represents a flexible and successful approach to obtain spatial and texture-relevant information. An MRF was also used by Vittitoe et al.^31^ to blend spatial and textural information using the potential functions that were parameterized with a probability distribution and the recursive conditional approach to classify each pixel of the chest X-ray fluoroscopic images optimally for the respective anatomic class. Furthermore, Ginneken et al.^8^ combined the rule-based approach with a shallow learning-based method, whereas the rule-based method uses prior information about the lung region. Also, the pixel classifier uses these attributes, the image intensities, and the entropy measurements to classify the X-ray image to their various categories, such as the lung regions and the non-lung regions. Shi et al.^32^ suggested an unsupervised technique to segment the lung regions. The authors used a CMC-based method with Gaussian kernels, and they achieved good results via the CMC’s flexible and robust mathematical modeling. Nevertheless, the CMC methods fail in medical image segmentation due to noise, artifacts, and illumination variations.

### Deep learning methods

Deep learning-based approaches are contrasted to shallow learning techniques, leverage dynamics, and hierarchical feature representations with several levels of abstraction. Deep learning methods significantly outperform the shallow learning techniques in many applications, but the most significant impediment to the practical deployment of deep learning is a lack of labeled data for training. In recent years the CNNs have been employed in medical field for diagnosing chest problems^33^. Kalinovsky et al.^34^ recently adopted the four-layered encoder–decoder architecture, which is called SegNet^35^, for the lung area segmentation. Novikov et al.^16^ adopted U-Net^22^ for multi-class segmentation in chest radiographs. Furthermore, Mittal et al.^36^ proposed LF-SegNet, a modified U-Net-based approach that incorporates a normalization mechanism and improves the up-sampling strategy for segmntation of lung fields in chest radiographs. However, these U-Net-based methods introduce outliers and holes inside the targeted structures, which were solved using a level-set method post-processing step.

Recently, amore complex CNN method such as Mask R-CNN^37^ is proposed by Wang et al.^38^ to segment and identify the lung field, heart, and clavicles in the chest X-ray images. However, this approach is computationally costly due to the excessive region proposals. Furthermore, Hwang et al.^5^ proposed a two-stage cascade network training approach, in which a network is trained using input chest X-rays in the first stage, and then both the input chest X-ray image and the output from the learned model in the first stage are fed into the second network. However, these approaches were not trained in an end to end manner. In addition, Peng et al.^39^ proposed a two-stage approach for the lung segmentation, where in the first step a Deep Belief Network and K-Nearest Neighbor is utilized to segment the lungs and then and improved principal curve and machine learning method is used for the refinement of the segmentation result. However, these types of techniques are computationally costly and necessitate a post-processing step to improve the output. In contrast, the proposed dual encoder–decoder method can be trained in the end-to-end manner. As a result, post-processing and pre-processing are not required.

**Figure 1:**
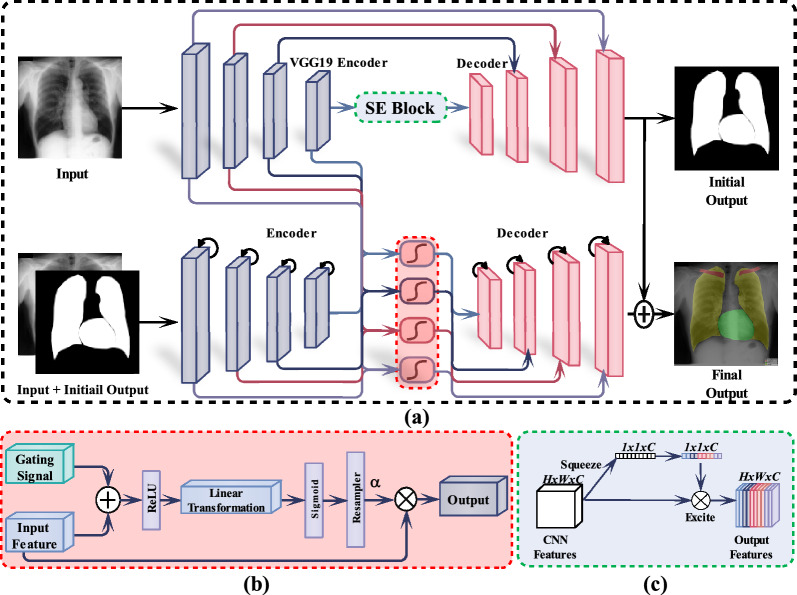
(**a**) The overall architecture of the proposed anatomical structures segmentation in the X-ray images, and (**b**) illustrates the attention-gating module in the red dotted lines. The attention coefficients computed in the AGM are used to scale the input features. The gating signal in the AGMs uses both the activations and the contextual information to identify the spatial regions. (**c**) The Squeeze-and-Excite (SE) block.

## Proposed method

Figure 1 shows the overview of the proposed framework. The proposed method contains two encoders and decoders. The image is first given to a pre-trained VGG19 encoder which of consists of highly connected convolutional and fully connected layers for better feature extraction. The extracted features from a pre-trained VGG19 encoder are then given to the SE block which pass the most relevant information to the first decoder for the generation of the initial binary segmentation mask. Furthermore, the initial binary segmentation mask and input image are coupled and fed to the second encoder to ensure that the produced initial binary segmentation mask can still be improved. The output features of the second encoder are passed through the AGMs, improving the quality of the feature maps. The AGMs focus on the regions of interest while maintaining the spatial resolution of the feature maps. The feature maps from both encoders are upsampled and passed to the second encoder which generate the final improved segmentation output. Moreover, the pre-trained VGG19 network can be replaced with other networks such as MobileNet^40^, ResNet^41^, DenseNet^42^ or EfficientNet^43^. Furthermore, incorporating DenseNet^42^ in our proposed framework obtained the best results.

### Dual encoder–decoder CNN architecture (DED-CNN)

#### Encoders

The first Deep Learning (DL) network employed a VGG19 encoder with successive convolution and max-pooling layers. The number of filters is doubled after the max-pooling layer, and the process is repeated four times. The fully connected layer is substituted with a single convolutional layer that serves as a network bottleneck, which separates the encoder and decoder. The Rectified Linear Unit (ReLU) activation function is used to introduce nonlinearity into the model. The Squeeze-and-Excite (SE)^44^block, which is shown in Fig. 1c, is then applied to enhance the feature map’s quality.

The second DL encoder is built from scratch. We utilize the prior segmentation mask estimated by the first encoder to guide the second encoder towards the instance of interest. The second network can learn appropriate boundaries of anatomical structures in chest X-ray images by emphasizing features derived from relatively coarse segmentation outputs. Furthermore the second encoder incorporates a recurrent residual convolutional layer (R2CL) in each step to increase the model’s capacity for integrating the context information. R2CL helps to develop a more deeper model and ensure better and stronger feature representation accumulation with respect to different time-steps. The R2CL is comprised of $3\times 3$ convolutions, which is repeated twice with recurrent connections. When the R2CL is employed, the number of feature maps increases, which results in a size reduction of about 50$\%$. Each convolution operation is followed by batch normalization (BN)^45^. The BN regularizes the model while reducing the internal covariant shift. Concatenation is used in the R2U-Net architecture to map the features from the encoding path to the decoding path. The residual network^46^ performs better when the R2CL operations are conducted in discrete time steps. As illustrated in Fig. 1a, we assume that $x_l$ is the input of the *l*th layer of the R2CL block. Where the pixel is localized at (*i*, *j*) in the input on the *k*th feature map. Furthermore, the network output $O_l$ is (*t*) at time step *t*. The output can be mathematically represented by the equation that is given below.

1 $$\begin{aligned} O^l_{ijk}(t) = (W^c_k)^T\times x^{c(i,j)}_l(t) +(W^r_k)^T\times x^{r(i,j)}_l (t-1)+b_k , \end{aligned}$$

wherein $x_l^{c(i,j)}$ and $x_l^{r(i,j)}$ are the standard convolutional layer inputs as well as the input for *l*th residual convolutional layer (RCL). $W_k^c$ and $W_k^r$ are the weights of the forward convolutional layer and RCL of the *k*th feature map, and $b_k$ is used as the bias. $O_{ijk}^l$ is given to the standard ReLU, which can be mathematically expressed using the equation that is given below.

2 $$\begin{aligned} F(x_l ,w_l)= f(O^l_{ijk}(t)) =0,O^l_{ijk}(t) \end{aligned}$$

Final outputs $x(l+1)$ of the recurrent convolutional unit is given to the residual unit, which can be formulated mathematically with the equation that is given below.

3 $$\begin{aligned} x_{l+1} = x_l + F(x_l ,w_l). \end{aligned}$$

**Figure 2:**
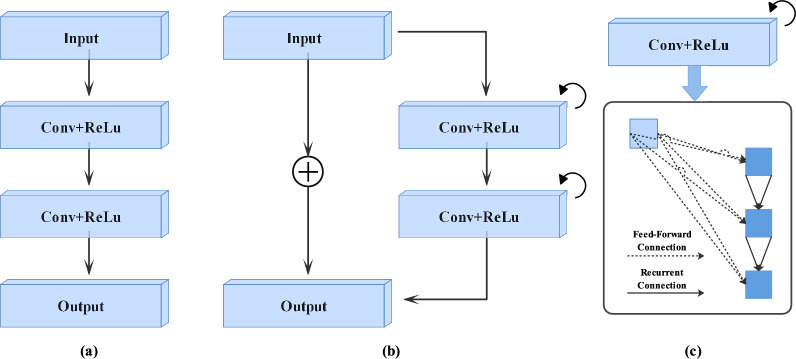
Convolutional and recurrent convolutional layers for different variants, which include (**a**) the forward convolutional layer, (**b**) the recurrent residual convolutional layer (R2CL), and (**c**) depicting the unfolded recurrent convolutional layer for T $=$ 2.

The inputs of R2CL are represented by $x_l$. The outputs $F(x_l,w_l)$ are used in the down-sampling and up-sampling layers in the encoder and decoder path of the proposed network. The successive sub-sampling or up-sampling layers use the final output $x_{l+1}$ as the input. The basic forward convolutional unit is shown in Fig. 2a, and the structure of the recurrent residual convolutional layer unit (R2CL) is shown in Fig. 2b. We deepen the R2CL block, which each comprise of two RCL units, to extract the features of the deeper layers. The RCL expansion strategy is represented in Fig. 2c, which is where the RCL is expanded to two-time steps $(T = 2)$.

#### Decoders

Each decoder block in the first DL network performs up-sampling on the given feature map via the transposed convolution, which effectively increases the size of the feature maps. Afterwards, the features maps of the encoder skip-connections are concatenated with the output feature maps. The skip-connections from the first encoder are used in the first decoder. Nevertheless, the skip-connections from both encoders are used in the second decoder, which allow the model to maintain spatial resolution and improve the quality maps of the output feature. In each step of the decoding path, the output of the R2CL unit from the preceding layer is up-sampled by the R2-Unit. The number of feature maps is reduced to half and size is doubled with each up-sampling process. The size of the feature map is restored to its original size of the input image at the last layer of the decoder. Furthermore, the BN is used in the up-sampling process to increase the proposed network’s stability and speed up its convergence during the training phase. The output of the BN is fed to the Attention Gating Modules (AGMs).

The second network uses the AGMs to optimize the previous encoder’s output features, which then merge them with the corresponding features in the decoder. Furthermore, the AGMs allow the model to emphasize the high-quality feature representations while preserving the spatial resolution and boosting the quality of the resulting feature maps. The additive attention map employed in AGMs is described in Fig. 1b. The attention values are computed for each pixel of all the input features $x_i^l$. To determine the attention regions in the feature map, the gating vector $g_i$ is applied to each pixel *i*. The following is the additive equation.

4 $$\begin{aligned} \alpha ^l_i = \sigma _2(\psi ^T(\sigma _1(W^T_x x^l_i + W^T_g g_i +b_g))+b_\psi , \end{aligned}$$

wherein $\sigma _1$ and $\sigma _2$ represent the ReLU and sigmoid activation. Furthermore, the weights of a linear transformation are represented as $W_x$ and $W_g$, and the biases are $b_g$ and $b_{\psi }$.  A linear transformation is used to minimize the number of learnable parameters and the computational complexity of the AGMs. In addition, we adjust the input features to match the size of the gating signal. To prevent the aliasing issue, grid resampling of the attention coefficients is performed using tri-linear interpolation. Our network incorporates the AGMs to emphasize the essential features to segment the clavicles, heart, and lungs. The coarse-scale information is used in the gating to disentangle the unnecessary and noisy responses in the skip connections, which boosts the model’s performance with the foreground pixel prediction. The output of the AGMs is the combination of the input feature map and the attention coefficient, which can be represented mathematically by the equation that is given below.

5 $$\begin{aligned} g_i = x^l_i \times \alpha ^l_i . \end{aligned}$$

Finally, the output is predicted using a sigmoid activation function.

The proposed framework was implemented using the Keras and TensorFlow libraries, and the models were trained from scratch. The model was initialized with random values during the training step. Adam was used to optimize the model with an initial learning rate (LR) of 1e-5 and a batch size of 8.

## Experiments and results

In this section, the datasets that were utilized to conduct the experiments are extensively discoursed, and the implementation procedure is described. In addition, the performance of the proposed framework to segment the anatomical structures in the chest fluoroscopic images is evaluated, and the results are discussed.

**Figure 3:**
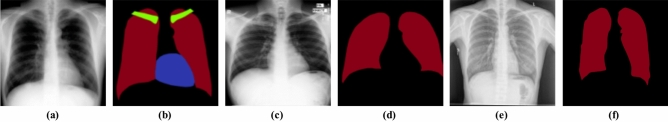
Input images and labels from three datasets: (**a**) radiographic X-ray image of the chest from JSRT dataset, (**b**) JSRT dataset labels of the lungs, clavicels and heart, (**c**) X-ray image of the chest from MCCXR dataset, (**d**) MCCXR dataset lungs label image, (**e**) chest X-ray image of SCXR dataset, and (**f**) SCXR dataset lungs label image.

### Datasets

We performed several experiments on three different datasets: MCCXR dataset^47^, the JSRT dataset^48^, and the SCXR dataset^47^ , as shown in Fig. 3. The JSRT dataset contains 247 posterior-anterior (PA) chest radiographs with a resolution of $2048\times 2048$ pixels and their ground-truth segmentation^8^ with a resolution of $1024\times 1024$ pixels. The MCCXR^47^ comprises of 138 fluoroscopic images with a resolution of $4020\times 4892$, which contains 80 normal subjects and 58 abnormal subjects, including tuberculosis, effusions, and military patterns gathered from Montgomery County’s tuberculosis screening program. The SCXR dataset^47^ was collected by Shenzhen No. 3 Hospital in Shenzhen, Guangdong providence, China. The SCXR dataset^47^ contain 326 normal X-rays images and 336 abnormal X-rays that show various manifestations of tuberculosis. Further, we have used NIH Chest X-ray dataset^49^ which contain both PA and AP views in cross-dataset evaluation. We have performed several experiments in cross-dataset evaluation setup on different datasets to validate the robustness and effectiveness of the proposed framework.

The JSRT dataset is initially split into five subsets. The five-folds cross-validation is then carried out with one fold being dedicated to testing, which includes 20$\%$ of all the available chest X-ray data. The remaining four folds are divided into training, which includes 90$\%$ the chest X-ray data, and the validation, which includes 10% of the chest X-ray data. For the SCXR and MCCXR datasets, we performed three fold cross-validation. For each fold, the total number of epochs was set to 100. An NVIDIA 1080Ti GPU was used to train each model. We compared the proposed model to the recent state of the art techniques, i.e., U-Net^22^, Linknet^50^, PSPNet^51^, Seg-Net^52^, Adaptive Scan^53^, X-Net+^54^, RX-Net+^54^, TVC^55^, TMI^16^, and SCIA^56^, For fair comparison, we normalized the image size as $256\times 256$ images and then trained the proposed model and the comparison methods.

### Performance metrics

We utilized the dice^57^, Intersection over Union (IoU)^58^, and the Hausdorff distance (HD)^59^ similarity coefficients to evaluate the proposed method and compare it to the SOTA approaches. For the pairwise comparison of the binary segmentation of the foreground with the ground truth, the DSC is a frequently used overlap metric. In formal terms, it is written using the equation that is given below.

6 $$\begin{aligned} Dice = \frac{2\times (A \cap B)}{A+B}, \end{aligned}$$

where the ground truth is A, and the predicated mask is B. The Dice coefficient varies from 0 to 1, and 1 indicates a complete overlap. In addition, we also utilized the Intersection over Union (IoU) metric to quantify the percent overlap between the target mask and our predicted output.

7 $$\begin{aligned} IoU = \frac{A \cap B}{|A| \cup |B|}. \end{aligned}$$

The HD is a distance measurement between two sets of points. HD is the maximum of the distances between any segmentation point and the nearest ground truth point.

8 $$\begin{aligned} HD = \max (d(A,B),d(B,A)), \end{aligned}$$

where *A* and *B* is the finite point set, the function *d*(*A*, *B*) is referred to as the directed HD from *A* to *B*, and *d*(*B*, *A*) is the distance from *B* to *A*.

**Table 1:** Comparative evaluation of the proposed method segmentation performance with the existing SOTA algorithms was reported on the JSRT dataset.

Methods	Clavicles				Lungs				Heart			
	IoU	IoU-FR	Dice	Dice-FR	IoU	IoU-FR	Dice	Dice-FR	IoU	IoU-FR	Dice	Dice-FR
Human^8^	–	0.896	–	0.945	–	0.946	–	0.972	–	0.878	–	0.935
X-Net+^54^	0.848	**0.874**	–	0.933	0.951	0.956	–	0.978	0.881	0.884		0.938
RX-Net+^54^	0.838	0.859	–	0.924	0.947	0.948	–	0.973	0.876	0.876	–	0.934
TVC^55^	–	–	–	–	–	0.951	–	0.975	–	0.893	–	0.943
SCIA^56^	0.863	–	0.926	–	**0.959**	–	**0.979**	–	0.899	–	0.947	–
TMI^16^	0.833	–	**0.929**	–	0.95	-	0.974	–	0.882	–	0.937	–
DED-CNN	0.86	–	0.909	–	0.954	–	0.974	–	0.906	–	0.949	–
DED-CNN*	0.868	–	0.91	–	0.955	–	0.976	–	**0.907**	–	**0.95**	–

The performance of the models was trained using full resolution (FR) (i.e., 2048 $\times$ 2048) and reported with IoU-FR and Dice-FR. The proposed method (*) represents the segmentation results of the model trained with the augmented dataset.

Significant values are in bold.

## Experimental results

This section presents the quantitative and qualitative results of the proposed method using three different datasets. In the quantitative results, the proposed framework performance of the multiclass segmentation and the single-class segmentation is compared with the different approaches using the JSRT dataset and the MCCXR and SCXR datasets. In the qualitative results, the visual results of the proposed method are demonstrated.

### Quantitative results

#### JSRT benchmark

Table 1 presents the comparison of the proposed work in terms of the segmentation performance with the existing approaches i.e X-Net+, RX-Net+, TVC, SCIA and TMI that are applied for all the anatomical structures of the X-ray images from the JSRT dataset, such as the heart, lungs, and clavicles. As we can see in Table 1, the proposed method outperformed the majority of the existing techniques. Our proposed method obtained the most significant results in terms of the IoU and dice score for the clavicles and heart segmentation. Additionally, our proposed method is comparably good for the lung segmentation. The RX-Net+ reported the lowest performance for the clavicles segmentation, which is 0.859 and 0.924 for the IoU and dice score, respectively. Another variant of the same method X-Net+ achieved slightly better results. However, this method uses an image size of $1024\times 1024$, which is computationally expansive and challenging to train a model. The proposed method also yielded higher dice and IoU scores than SCIA for the clavicles and heart and comparable dice and IoU scores for the lungs. Although SCIA achieved slightly better results on the lungs than ours, SCIA employed a level set approach to post-process the output segmentation to remove the artifacts, which is computationally costly. In contrast, no post-processing is performed in the proposed method for the improvement of the output segmentation.

**Table 2:** An overview of the results based on the IoU and the Hausdorff distance for each architecture and three organs, which include the clavicles, heart, and lungs.

Methods	Organs	IoU					Hausdorff distance				
		Mean	Std	Median	Min	Max	Mean	Std	Median	Min	Max
TMI^16^	Clavicles	0.833	0.833	0.843	0.639	0.905	22.39	10.721	20.18	6.031	72.764
	Heart	0.869	0.869	0.894	0.511	0.955	50.245	32.378	40.464	13.867	195.971
	Lungs	0.951	0.951	0.957	0.842	0.972	56.795	40.207	42.804	13.176	229.373
RX-Net+ ^54^	Clavicles	0.86	0.86	0.874	0.661	0.929	20.047	10.479	17.202	7.066	70.187
	Heart	0.889	0.889	0.905	0.738	0.961	38.007	18.074	34.35	13.15	99.823
	Lungs	**0.955**	0.955	0.961	0.889	0.976	45.146	30.254	36.291	11.338	167.7
DED-CNN	Clavicles	**0.868**	0.868	0.873	0.706	0.935	38.694	3.275	38.849	31.118	46.342
	Heart	**0.907**	0.907	0.916	0.767	0.961	**37.186**	3.557	37.2	30.044	45.587
	Lungs	**0.955**	0.955	0.958	0.881	0.972	**37.419**	3.535	37.464	28.347	44.961

The bolded scores are the best-performing networks on the JSRT dataset according to each column’s metric.

Table 2 presents the results of the comparison models trained on a single-class dataset. Similar to the results on the multi-class, DED-CNN outperformed the RX-Net+ and TMI. DED-CNN achieved 0.868 IoU for clavicle segmentation, whereas the TMI had the lowest IoU of 0.833. Also, the RX-Net+ showed a slightly worse IoU than our proposed model by achieving a 0.86 IoU. The proposed method obtained a 0.907 IoU for heart segmentation, whereas the RX-Net+ and the TMI scored 0.889 and 0.869 IoU, respectively. The RX-Net+ and the proposed DED-CNN achieved a similar IoU score of 0.955. However, the proposed method outperformed the TMI for lung segmentation. In terms of the HD, the proposed DED-CNN outperforms both the RX-Net+ and TMI for the heart and lungs by achieving 37.186 and 37.419 of HD, respectively. For the clavicles, the RX-Net+ achieved a lower HD than our proposed method.

#### MCCXR benchmark

We also compared our proposed model in terms of the lung segmentation with the existing methods for the MCCXR dataset, which are presented in Table 3. The proposed DED-CNN outperforms the other existing approaches. The proposed DED-CNN attained a dice score of 97.67$\%$, and an IoU of 95.48$\%$. The proposed model achieved higher scores than the baseline UNet by 2.2$\%$ and 2.92$\%$ in terms of the dice score and IoU. In addition, our model also achieved a higher score when it was compared with the PSPNet model.

**Table 3:** Analysis of the lung segmentation results in the terms of the dice score and the IoU score using the MCCXR dataset.

Methods	Dice	IoU
Fuzzy C-mean^60^	0.9580	0.9350
Atlas-based NR^12^	0.9600	0.9410
Deformation-tolerant^61^	0.9230	0.8620
Adaptive scan^53^	0.9592	0.9216
U-NET^22^	0.9547	0.9256
SegNet^52^	0.8418	0.8914
Linknet^50^	0.9687	0.9397
PSPNet^51^	0.3889	0.2443
DED-CNN	**0.9767**	**0.9548**

The bolded values demonstrate that the proposed approach performed better in terms of the metric presented in each column.

#### SCXR benchmark

The proposed method is also applied for lung segmentation on the SCXR dataset. As shown in Table 4, we achieved better results than the existing methods. As we can see in Table 4, the proposed DED-CNN outperforms the other methods by achieving a dice score and an IoU of 97.67$\%$ and 95.48$\%$, respectively. These results show that the proposed method outperformed the baseline UNet by 2.2$\%$ dice score and 2.92$\%$ IoU. Moreover, PSPNet showed the lowest performance when it was compared to the achieved results from the proposed method.

**Table 4:** Comparision of the proposed method with the other exisiting models in terms of lung segmentation using the SCXR database.

Methods	Dice	IoU
U-NET^22^	0.9532	0.9119
Linknet^50^	0.9491	0.9044
PSPNet^51^	0.6685	0.5420
FPN^62^	0.9023	0.8328
DED-CNN	**0.9568**	**0.9184**

The bolded values demonstrate that the proposed method performs better in terms of the metric presented in each column.

To further confirm the significance of the proposed DED-CNN, we compared it with the existing deep learning methods in the literature using three standard benchmark datasets. We avoided the traditional methods, because our main aim was to compare the proposed model with the deep learning based approaches for lung segmentation. Table 5 presents the comparison results of the proposed method and the existing deep CNN architectures for lung segmentation. As we can see in Table 5, the proposed method achieved dice scores of 97.60$\%$, 97.67$\%$, and 95.68$\%$, and it achieved IoU scores of 95.50$\%$, 95.48$\%$, and 91.84$\%$ for the JSRT, MCCXR, and SCXR datasets, respectively. We compared these results with the U-Net model, which indicate that the proposed model increases the dice score by +0.2$\%$, +2.2$\%$, and +0.36$\%$, and IoU score by +3.3$\%$, +2.92$\%$, and +0.65$\%$ for the JSRT, MCCXR, and SCXR datasets, respectively. For the JSRT dataset, the proposed DED-CNN surpasses the dice score by +0.4$\%$ and the JI score by +0.9$\%$ when it is compared to the gold standard^8^. These results indicate that the proposed method outperformed the recently published segmentation approaches in terms of the dice and IoU scores using the three datasets.

**Table 5:** Detailed comparison of the proposed method with the existing models using three different datasets.

Methods	Datasets	Method description	Evaluation	
			Dice	IoU
Human^8^	JSRT	Annotated by humans	0.9720	0.9460
	JSRT		0.9740	0.9220
Ronneberge et al.^22^	MCCXR	U-Net:encoder–decoder CNN	0.9547	0.9256
	SCXR		0.9532	0.9119
Suza et al.^23^	MCCXR	AlexNet for patch classifcation and ResNet18 based reconstruction	0.9400	0.8800
Novikov et al.^16^	JSRT	FCN Inverted-Net	0.9685	0.9490
Lei et al.^56^	JSRT	GAN based segmentation	0.9747	0.9508
**DED-CNN**	JSRT	Dual encoder–decoder structure	**0.9760**	**0.9550**
	MCCXR		**0.9767**	**0.9548**
	SCXR		**0.9568**	**0.9184**

The bolded values represent the best performances.

#### Qualitative results

**Figure 4:**
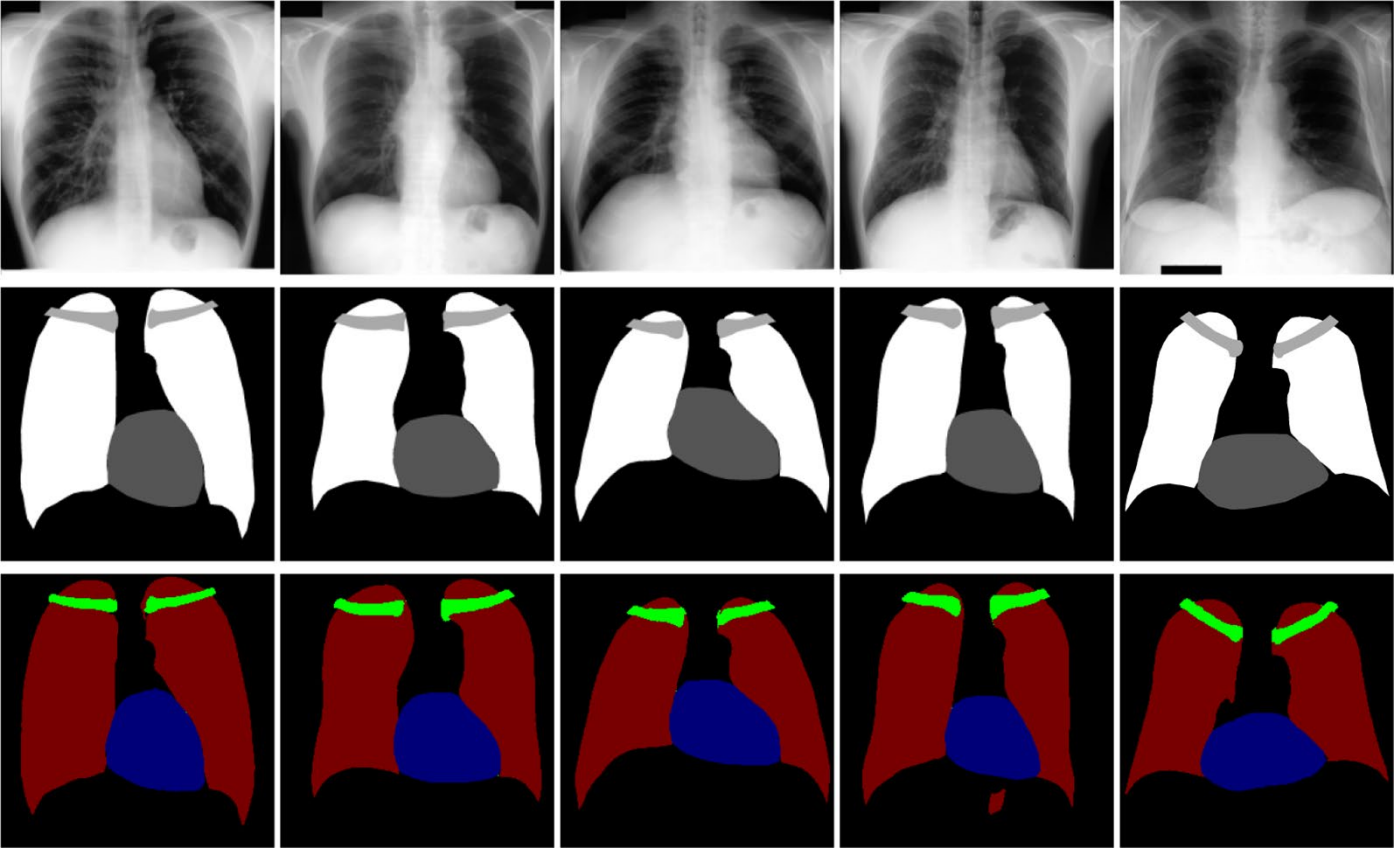
Qualitative results of our proposed method on a test sample from the JSRT dataset. From top to bottom: inputs, groundtruth labels, and the segmentation prediction.

**Figure 5:**
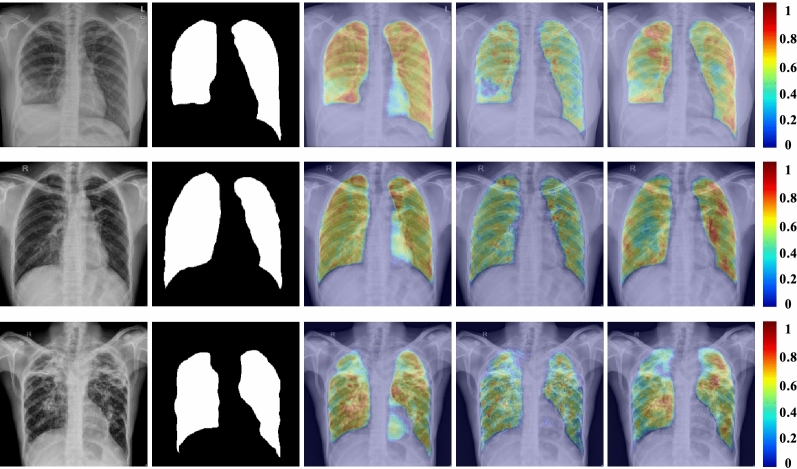
Class activation map (CAM) for the lung region. From left to right: inputs, groundtruth labels, U-Net CAM, Linknet CAM, and the proposed method CAM.

To confirm the qualitative performance and further assess the efficiency of the proposed method, the visual results of the different anatomical structures segmentation are demonstrated in Fig. 4. The class activation maps (CAMs) strategy was initially adopted to interpret classification models; however, Vinogradova et al.^63^ used the CAMs for the interpretation of semantic segmentation networks. We analyzed the feature’s activation here and employed CAMs to generate the heat maps to visualize the most activated areas in the X-ray images^64^. In addition, the last layer of the trained model is retrieved to construct the CAMs. By taking the weighted sum of the feature maps with their associated weights, we obtained a map, which is denoted as $C_m$, of the most important features, which is used to identify the image pixel as the lung region. The map $C_m$ can be formally stated using the equation that is given below.

9 $$\begin{aligned} C_m = \sum _k w_{c,k},f_k, \end{aligned}$$

where $f_k$ is the last feature map, and $w_{c,k}$ is weight of the final classification layer. We identify the essential features, which are utilized by the model to predict the lung region and by up-scaling the map $C_m$ to the image’s dimensions and overlaying the input image. Figure 5 shows various examples of the lung segmentation task using the CAMs. As shown in Fig. 5, the CAMs that are employed in our proposed method produced more activation on specific lung regions, whereas the U-Net CAMs have some outliers that were triggered on the right lung’s bottom side. In addition, the Linknet CAMs have less activation on the left lung when it was compared to the proposed CAMs.

## Discussion

The popularity of the CADs among physicians and healthcare professionals is increasing daily. Therefore, it has become vital to provide a stable and reliable framework for chest radiographs that can be constantly evaluated and used for possible medical diagnostics. This study provides a new method for autonomous multi-organ and lung area segmentation in the chest X-rays. The proposed method incorporates a dual encoder–decoder CNN architecture with an initial encoder, which is a pre-trained VGG19 network. In this model, the attention gates and recurrent residual convolutional blocks are utilized instead of the regular convolutional blocks in the second network. The extensive experimental evaluation affirmed that the proposed dual encoder–decoder CNN architectures produced the best results for anatomical structure segmentation compared to the state-of- art methods.

**Table 6:** Detailed comparison of the proposed method in the cross-dataset setting. The proposed method includes two encoder-decoder networks, referred to as Network-1 and Network-2, respectively.

Methods	Train dataset	Test dataset	Dice	IoU
DED-CNN	JSRT	SCXR	**0.9525**	**0.9108**
DED-CNN w/o SE	JSRT	SCXR	0.9381	0.8855
DED-CNN w/o AGM	JSRT	SCXR	0.9399	0.8869
Network-1	JSRT	SCXR	0.9416	0.8913
Network-2	JSRT	SCXR	0.9480	0.9014
DED-CNN	SCXR	MCCXR	**0.9597**	**0.9228**
DED-CNN w/o SE	SCXR	MCCXR	0.9478	0.9013
DED-CNN w/o AGM	SCXR	MCCXR	0.9550	0.9142
Network-1	SCXR	MCCXR	0.9495	0.9068
Network-2	SCXR	MCCXR	0.9559	0.9167
DED-CNN	SCXR+MCCXR	NIH	**0.9168**	**0.8470**
DED-CNN w/o AGM	SCXR+MCCXR	NIH	0.9148	0.8436
Network-1	SCXR+MCCXR	NIH	0.9051	0.8383
Network-2	SCXR+MCCXR	NIH	0.9081	0.8433

The bolded values represent the best performances.

**Figure 6:**
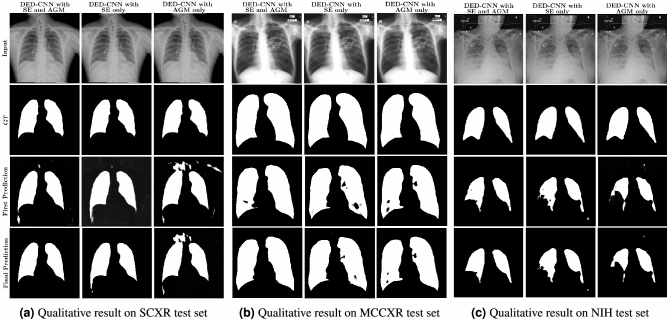
Qualitative results of the proposed DED-CNN in the cross-dataset setting where all the models are trained on different dataset and tested on different dataset. From left to right: the first row is the input image, the second row represent the ground truths, the third row is the prediction result of the first encode-decoder model in DED-CNN framework and the last row represent the results of the second encoder–decoder model in the DED-CNN framework. All the models are trained and test in cross dataset configuration.

**Table 7:** Cross-dataset qualitative comparisons of the component of the proposed approach.

Methods	Pre-trained model	Train dataset	Test dataset	Dice	IoU
DED-CNN	VGG-19^65^	JSRT	SCXR	0.9525	0.9108
	ResNet-50^41^			0.9540	0.9160
	DenseNet-121^42^			**0.9565**	**0.9166**
	MobileNet-v2^40^			0.9491	0.9073
	EfficientNet^43^			0.9508	0.9096
DED-CNN	VGG-19^65^	SCXR	MCCXR	0.9597	0.9228
	ResNet-50^41^			0.9599	0.9236
	DenseNet-121^42^			**0.9610**	**0.9297**
	MobileNet-v2^40^			0.9423	0.9183
	EfficientNet^43^			0.9537	0.9213
DED-CNN	VGG-19^65^	SCXR+MCCXR	NIH	0.9168	0.8470
	ResNet-50^41^			0.9204	0.8530
	DenseNet-121^42^			**0.9334**	**0.8752**
	MobileNet -v2^40^			0.9287	0.8672
	EfficientNet^43^			0.9213	0.8557

The values in bold represent the best performances.

The results showed in Fig. 4 confirmed the effectiveness of our proposed method in regards to segmenting several organs in the chest X-ray fluoroscopic radiographs. Furthermore, the results provided in Fig. 5 demonstrate that our technique can segment the heart, clavicles, and lungs in severely aberrant structures, which makes it suitable for clinic diagnostics. Furthermore, we performed lung segmentation generalization across datasets in order to evaluate all possible dataset combinations. Table 6 shows the dice and IoU scores for the proposed DED-CNN. The performance of the DED-CNN is higher to that of the model that was trained using only AGM or SE blocks on test datasets. Furthermore, the Fig. 6 demonstrate that the qualitative results are consistent with our quantitative results presented in Table 6 for cross dataset testing configuration. Moreover, the performance of the proposed method with various pre-trained models is shown in Table 7. When we employed the DenseNet-121 as a pre-trained model in our proposed approach, we obtained the best dice and IoU score in cross-dataset settings compared to VGG-19, ReseNet-50, MobileNetv2 and EfficientNet pre-trained models.

Our proposed approach has notable advantages over SCIA and TMI since it is an “end-to-end” method and has robust accuracy performances. There is no pipeline that necessitates a significant amount of image pre and post-processing or registration, which leads to a noticeable decrease in performance time. Additionally, by utilizing the first network output in the second network, where we combine the input image and output segmentation map to guide the second network, our approach effectively lowers outliers and misclassification. In contrast to SCIA and TMI, the proposed method makes use of a pre-trained model, maximizing its generalizability. Additionally, using a pre-trained model is advantageous when there is limited training data.

Despite the outstanding performance of the proposed model in various settings, we illustrated some flaws and constraints that should be carefully considered. First and foremost, the proposed system only accomplishes the segmentation task. Second, this approach is computationally expensive because it uses a dual encoder and decoder. We will integrate additional downstream tasks in future studies, such as pneumonia classification to provide a more comprehensive pipeline for the CAD of chest radiographs. In addition, we also aim to increase the speed of the proposed method by reducing the model parameters using pruning.

## Conclusion

In this study, we presented a deep learning-based framework that can effectively identify anatomical structures, which include the lungs, heart, and clavicles, in chest X-ray images. A dual encoder–decoder network is notably employed, which can iteratively refine the output of the first network by fusing it with the input image and passing it through the second network to identify the anatomical structures in the X-ray images. Furthermore, the first encoder–decoder incorporates the use of a pre-trained VGG19 network, which allows the proposed framework to be efficiently trained using limited datasets. In addition, it also allows the proposed model to extract essential features to enhance the anatomical structure segmentation. In the second encoder–decoder, we integrated the input image with a segmentation mask to guide the network and focus on the essential features and avoid the outliers, which permits the proposed network to segment the anatomical structures effectively. Furthermore, instead of the standard convolutional layers, we employed the R2CL and AGMs, which enable the proposed framework to focus on the regions of interest simultaneously and improve the feature maps. The proposed method accurately extracts information from the first encoder–decoder network and integrates it with the second encoder–decoder network to provide precise segmentation of the anatomical structures, which will assist physicians with diagnosing various pulmonary and cardiac diseases.

In the future, the performance of the anatomical structures segmentation framework will be enhanced by utilizing synthetically generated samples using generative adversarial networks. Additionally, downstream tasks, such as pneumonia and covid classification, will be included to provide a more comprehensive approach for the CADs of chest radiographs. Finally, a more sophisticated segmentation and classification method will be investigated for more accurate and reliable anatomical structure segmentation and diseases classification.

## Data Availability

The JSRT dataset used in this study is published by the Japanese Society of Radiology Technology (JSRT) and is accessible at http://db.jsrt.or.jp/eng.php, The Montgomery dataset (MCCXR) used in this study is published by the U.S. National Institute of Health and is accessible at https://academictorrents.com/details/ac786f74878a5775c81d490b23842fd4736bfe33. The Shenzen dataset (SCXR) images are available on https://www.kaggle.com/datasets/raddar/tuberculosis-chest-xrays-shenzhen and Shenzen dataset (SCXR) masks are accessable at https://www.kaggle.com/datasets/yoctoman/shcxr-lung-mask. The NIH dataset used in this work is publicly available on https://github.com/rsummers11/CADLab/tree/master/Lung_Segmentation_XLSor.
