# Author Correction: A deep learning based dual encoder–decoder framework for anatomical structure segmentation in chest X-ray images

**DOI:** 10.1038/s41598-023-41661-w

**Published:** 2023-09-04

**Authors:** Ihsan Ullah, Farman Ali, Babar Shah, Shaker El-Sappagh, Tamer Abuhmed, Sang Hyun Park

**Affiliations:** 1 Department of Robotics and Mechatronics Engineering, Daegu Gyeonbuk Institute of Science and Engineering (DGIST), Daegu, 42988 South Korea; 2 Department of Computer Science and Engineering, School of Convergence, College of Computing and Informatics, Sungkyunkwan University, Seoul, 03063 South Korea; 3 College of Technological Innovation, Zayed University, 19282 Dubai, United Arab Emirates; 4 Faculty of Computer Science and Engineering, Galala University, Suez, 435611 Egypt; 5 Information Systems Department, Faculty of Computers and Artificial Intelligence, Benha University, Banha, 13518 Egypt; 6 Department of Computer Science and Engineering, College of Computing and Informatics, Sungkyunkwan University, Suwon, 16419 South Korea

Correction to: *Scientific Reports* https://doi.org/10.1038/s41598-023-27815-w, published online 16 January 2023

In the original version of this Article, Farman Ali was incorrectly affiliated with ‘Department of Software, Sejong University, Seoul, 05006, South Korea’. The correct affiliation is listed below.

‘Department of Computer Science and Engineering, School of Convergence, College of Computing and Informatics, Sungkyunkwan University, Seoul 03063, South Korea.’

The original Article has been corrected.

